# Combined digestive endoscopy and laryngoscopy for successful management of an early-stage epiglottic carcinoma

**DOI:** 10.1055/a-2761-0100

**Published:** 2026-01-08

**Authors:** Xun-Mei Duan, Yu Bao, Ye-Han Zhou, Zhen-Ming Zhang

**Affiliations:** 1 92293 Department of Endoscopy Center, Sichuan Clinical Research Center for Cancer, Sichuan Cancer Hospital and Institute, Sichuan Cancer Center, Affiliated Cancer Hospital of University of Electronic Science and Technology of China, Chengdu, China; 2 92293 Department of Pathology, Sichuan Clinical Research Center for Cancer, Sichuan Cancer Hospital and Institute, Sichuan Cancer Center, Affiliated Cancer Hospital of University of Electronic Science and Technology of China, Chengdu, China

**Fig. 1:**
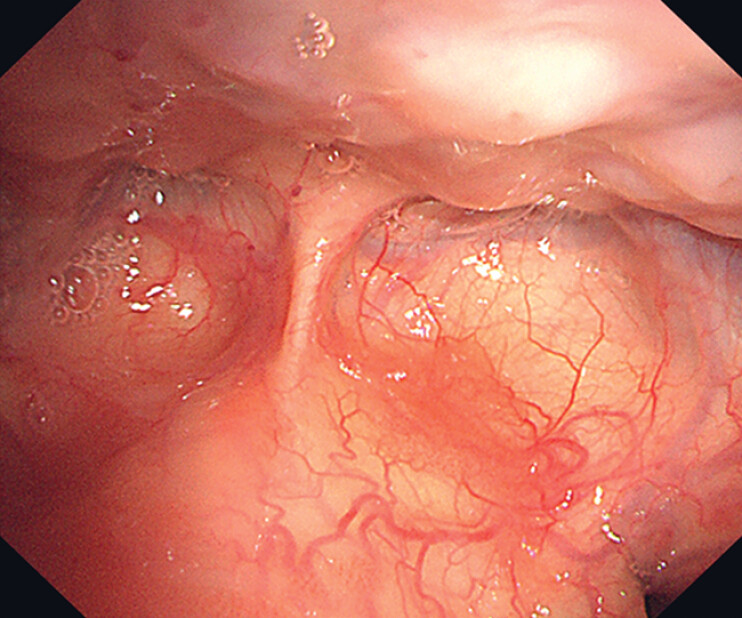
The lesions in the epiglottis were flat (0-IIb) and slightly reddish under white light laryngoscopy.

**Fig. 2:**
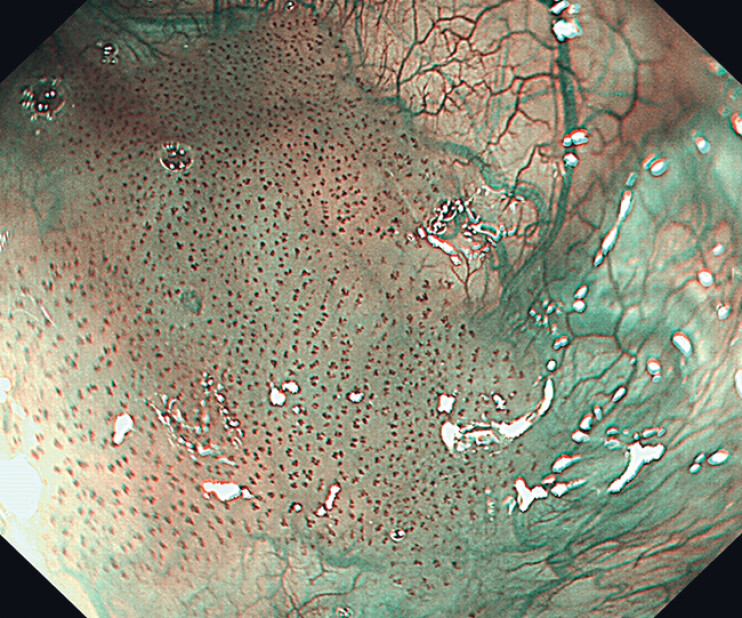
The appearance of the neoplastic lesions under narrow-band imaging.

A 70-year-old man with a history of resected esophageal carcinoma was hospitalized due to the discovery of several lesions on the surface of the epiglottis during follow-up laryngoscopy. Laryngoscopy revealed multifocal and irregular lesions scattered on the lingual surface of the epiglottis. These flat (0-IIb), reddish lesions displayed B1-type intraepithelial papillary capillary loops under narrow-band imaging ( Fig. 1 , Fig. 2 ). A tumor biopsy revealed in situ squamous cell carcinoma. Meanwhile, enhanced computed tomography indicated an absence of lymph node involvement.

**Fig. 3:**
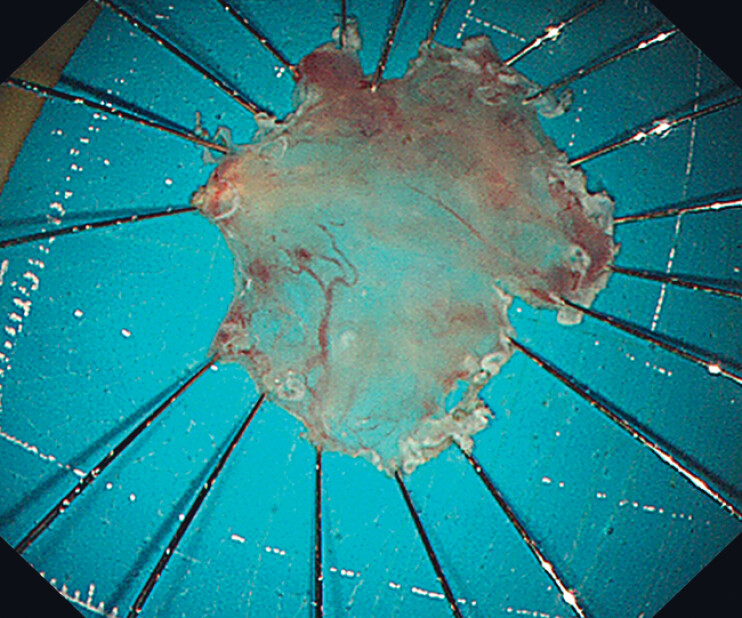
The resected specimen of the lesions.

**Fig. 4:**
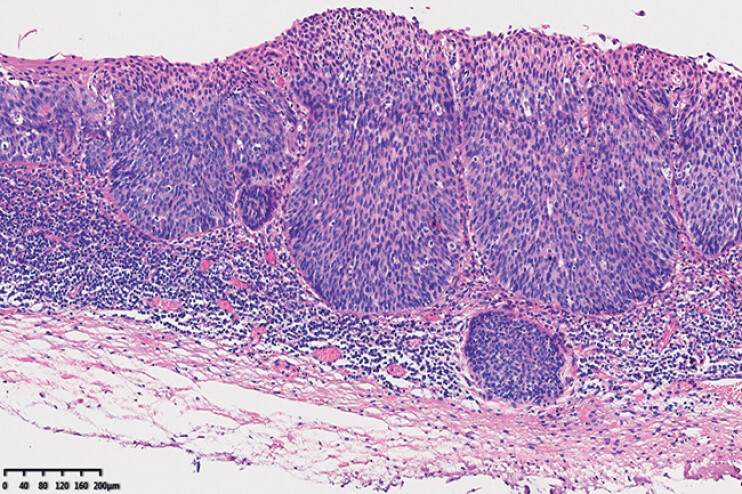
Histopathological analysis confirmed the presence of squamous cell carcinoma that locally invaded the lamina propria.

**Fig. 5:**
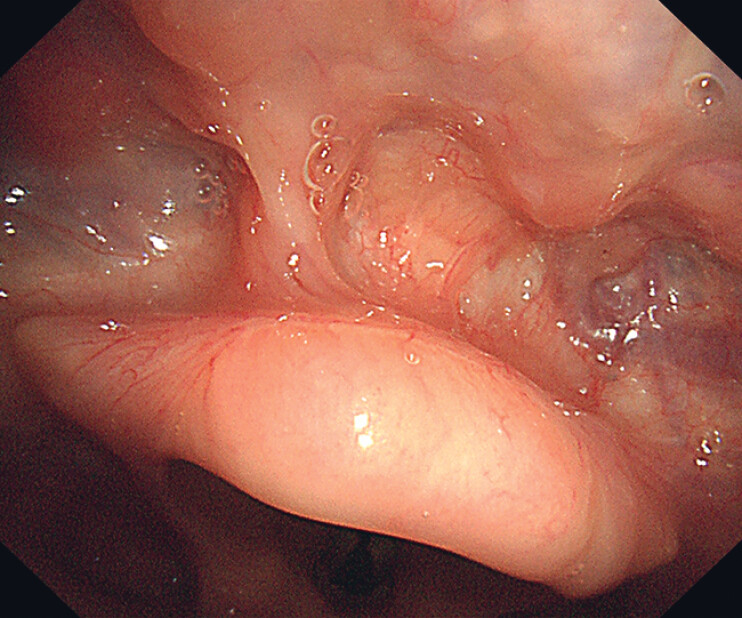
Follow-up laryngoscopy showing that the surgical wound has completely healed with scar formation.

Endoscopic submucosal dissection (ESD) was performed under general anesthesia induced via nasal intubation ( Video 1 ). A therapeutic gastroscope was used for the procedure. The constrained anatomical space of the epiglottic vallecula necessitated the use of a snare traction approach to facilitate submucosal dissection in challenging areas. Subsequently, en bloc resection of the lesions was achieved ( Fig. 3 ). No adverse events were observed during or after the procedure. Histopathological analysis confirmed the local invasion of squamous cell carcinoma into the lamina propria and showed that R0 resection was achieved ( Fig. 4 ). A follow-up laryngoscopy conducted 3 months post-ESD revealed complete healing of the surgical site ( Fig. 5 ), and subsequent routine follow-ups over 46 months detected neither local recurrence nor lymphadenopathy.

Combined digestive endoscopy and laryngoscopy for the successful management of an early-stage epiglottic carcinoma.Video 1

In this case, the epiglottic mucosal lesions were initially overlooked on gastroscopy; however, they were subsequently detected by laryngoscopy. This finding highlights the complementary diagnostic value of laryngoscopy alongside gastroscopy in screening high-risk populations for pharyngeal cancer 1 . Complete endoscopic resection via digestive endoscopy was then performed, demonstrating its essential role in the treatment of early-stage pharyngeal cancer 2 3 . Post–ESD follow-up was conducted via laryngoscopy under local anesthesia, providing a well-tolerated and patient-friendly approach. This case highlights the clinical significance of an integrated strategy combining digestive endoscopy and laryngoscopy for the comprehensive management of early pharyngeal cancer, supporting its broader adoption in clinical practice.

Endoscopy_UCTN_Code_TTT_1AO_2AG_3AD
